# In-Silico Mining of the Toxins Database (T3DB) towards Hunting Prospective Candidates as ABCB1 Inhibitors: Integrated Molecular Docking and Lipid Bilayer-Enhanced Molecular Dynamics Study

**DOI:** 10.3390/ph16071019

**Published:** 2023-07-18

**Authors:** Mahmoud A. A. Ibrahim, Khlood A. A. Abdeljawaad, Alaa H. M. Abdelrahman, Peter A. Sidhom, Ahmed M. Tawfeek, Gamal A. H. Mekhemer, Mohamed K. Abd El-Rahman, Eslam Dabbish, Tamer Shoeib

**Affiliations:** 1 Computational Chemistry Laboratory, Chemistry Department, Faculty of Science, Minia University, Minia 61519, Egypt; 2 School of Health Sciences, University of KwaZulu-Natal, Westville Campus, Durban 4000, South Africa; 3 Department of Pharmaceutical Chemistry, Faculty of Pharmacy, Tanta University, Tanta 31527, Egypt; 4 Chemistry Department, College of Science, King Saud University, Riyadh 11451, Saudi Arabia; 5 Department of Chemistry and Chemical Biology, Harvard University, 12 Oxford Street, Cambridge, MA 02138, USA; 6 Department of Chemistry, The American University in Cairo, New Cairo 11835, Egypt

**Keywords:** MDR, ABCB1 transporter, Toxin and Toxin-Target Database (T3DB), docking computations, MD simulations

## Abstract

Multidrug resistance (MDR) is one of the most problematic issues in chemotherapeutic carcinoma therapy. The ABCB1 transporter, a drug efflux pump overexpressed in cancer cells, has been thoroughly investigated for its association with MDR. Thus, discovering ABCB1 inhibitors can reverse the MDR in cancer cells. In the current work, a molecular docking technique was utilized for hunting the most prospective ABCB1 inhibitors from the Toxin and Toxin-Target Database (T3DB). Based on the docking computations, the most promising T3DB compounds complexed with the ABCB1 transporter were subjected to molecular dynamics (MD) simulations over 100 ns. Utilizing the MM-GBSA approach, the corresponding binding affinities were computed. Compared to ZQU (calc. −49.8 kcal/mol), Emamectin B1a (T3D1043), Emamectin B1b (T3D1044), Vincristine (T3D4016), Vinblastine (T3D4017), and Vindesine (T3D2479) complexed with ABCB1 transporter demonstrated outstanding binding affinities with Δ*G*_binding_ values of −93.0, −92.6, −93.8, −92.2, and −90.8 kcal/mol, respectively. The structural and energetic investigations confirmed the constancy of the identified T3DB compounds complexed with the ABCB1 transporter during the 100 ns MD course. To mimic the physiological conditions, MD simulations were conducted for those identified inhibitors complexed with ABCB1 transporter in the presence of a POPC membrane. These findings revealed that Emamectin B1a, Emamectin B1b, Vincristine, Vinblastine, and Vindesine are promising ABCB1 inhibitors that can reverse the MDR. Therefore, subjecting those compounds to further in-vitro and in-vivo investigations is worthwhile.

## 1. Introduction

**Figure 1:**
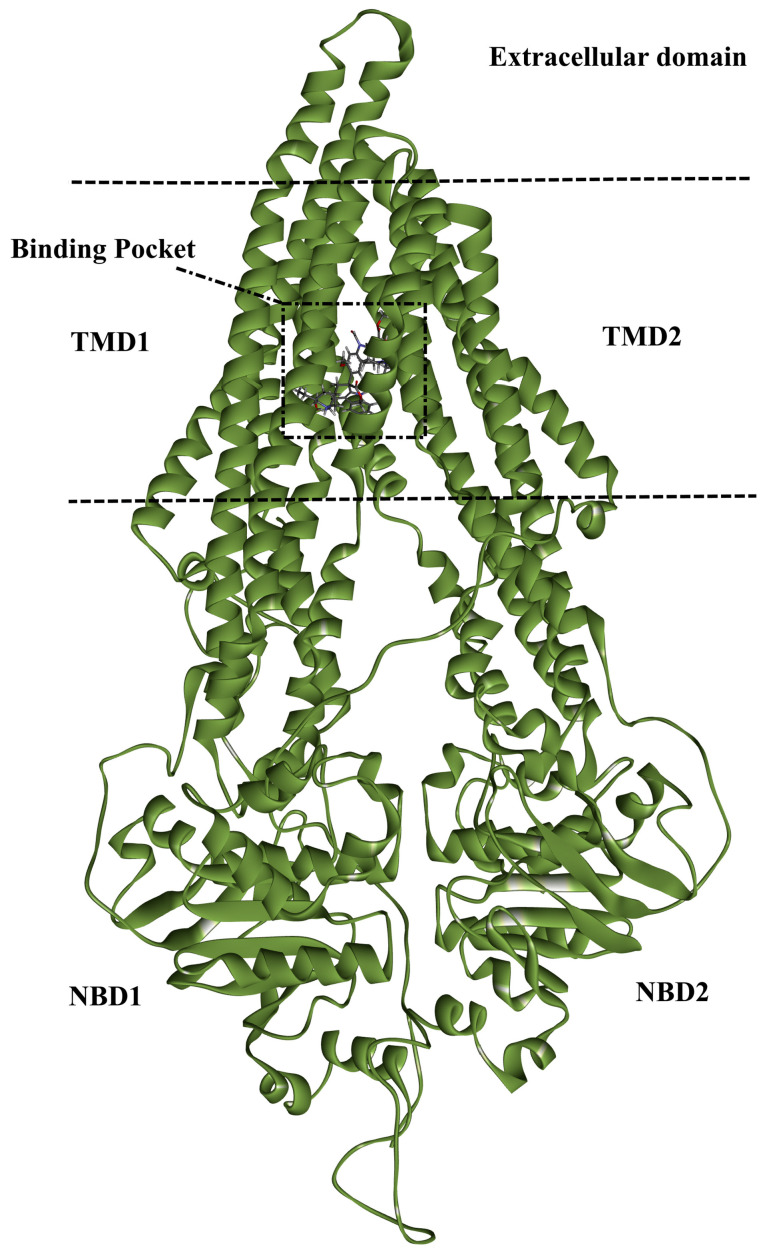
Schematic representation of ABCB1 transporter.

Chemotherapy is one of the most efficient treatment approaches for patients with advanced or metastatic cancers [1]. However, a critical problem in treating cancer is the multidrug resistance (MDR) phenomenon that emerges when cancer cells simultaneously develop resistance to a variety of anticancer therapeutics [2]. Cancer cells become multidrug-resistant cells by using a variety of resistance development pathways, including efflux pumps, apoptosis control, transcriptional regulation, autophagy control, and histone modifications [3]. Indeed, the augmented efflux of chemotherapy regulated through transmembrane transporters is the most common one [4,5]. These transporters, known as ATP-binding cassette (ABC) transporters, consist of 48 members and are found in the plasma membranes [6]. The ABC family is divided into seven subfamilies named ABC-A to ABC-G according to their sequence similarity [7]. An ATP hydrolysis process is required for those transporters to produce energy to efflux various foreign substances through the cell membrane [8,9]. The ABCB1 transporter, an ABC-B subfamily member, is well known for being a key player in the development of MDR in cancer cells [10]. ABCB1 is a membrane protein with a molecular weight of 170 kDa and was first reported in 1976 as an overexpressed protein in a cell line that was resistant to Colchicine [11]. The ABCB1 transporter is typically expressed in the epithelium of many various tissues, like the blood-brain barrier (BBB), placenta, and kidney [12]. The ABCB1 structure consists of two transmembrane domains (TMDs) and two nucleotide-binding domains (NBDs) [13]. The drug-binding pocket is located at the interface of the two TMDs of the ABCB1 transporter as Figure 1 demonstrates.

It is well known that inhibiting the ABCB1 transporter’s efflux activity can reverse the MDR phenomenon in cancer cells. The MDR phenomenon mediated by the ABCB1 transporter can be reversed in cancer cells by inhibiting its efflux function [14]. Several inhibitors experimentally demonstrated promising efficiency against the ABCB1 transporter; however, most of them failed in clinical trials because of their poor selectivity, insufficient efficiency, or significant cytotoxicity [15]. Therefore, identifying novel ABCB1 inhibitors is urgently needed to reverse the MDR in cancer cells.

**Figure 2:**
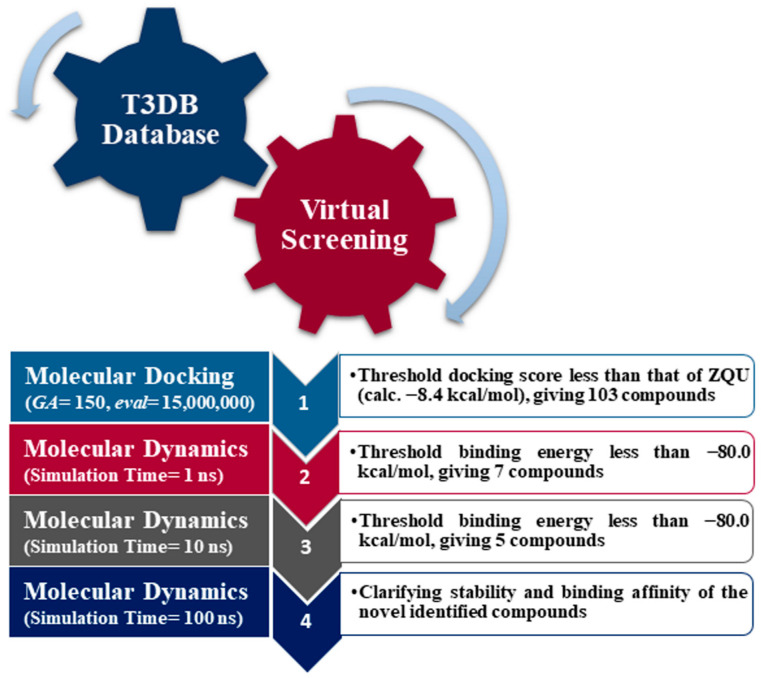
Flowchart diagram of the utilized computational methods employed in steps 1-4 displayed in the arrows for identifying potential ABCB1 inhibitors from the T3DB database.

Toxins can be used as chemotherapeutic drugs to treat many illnesses when they are not harmful to humans [16]. It has been documented that eleven drugs approved by the Food and Drug Administration (FDA) are identified as toxins, such as Lixisenatide, Exanta, Exenatide, and Ziconotide [17]. Drug repurposing can reduce the trial steps, time, and cost for the medicine to reach the market. Recent research has concentrated on assessing the effectiveness of numerous repurposed medications as potential anticancer treatments [18,19]. Thus, the Toxin and Toxin-Target Database (T3DB), containing more than 3600 compounds, is a valuable source for hunting potential ABCB1 inhibitors [20]. Herein, the T3DB database was filtrated against the ABCB1 transporter utilizing AutoDock4.2.6 software. According to the docking scores, the top promising T3DB compounds as ABCB1 inhibitors were subsequently subjected to molecular dynamics (MD) simulations. Additionally, throughout 100 ns MD simulations, in the absence and presence of a lipid bilayer membrane, the binding energies and stability of the potent ABCB1 inhibitors were further examined. Figure 2 shows an illustrative representation of the computational methods utilized for the T3DB database filtration. The outcomes of the current work may offer valuable insights into the suitability of the identified T3DB compounds as prospective clinical candidates for cancer treatment.

## 2. Results and Discussion

### 2.1. In-Silico Protocol Validation

**Figure 3:**
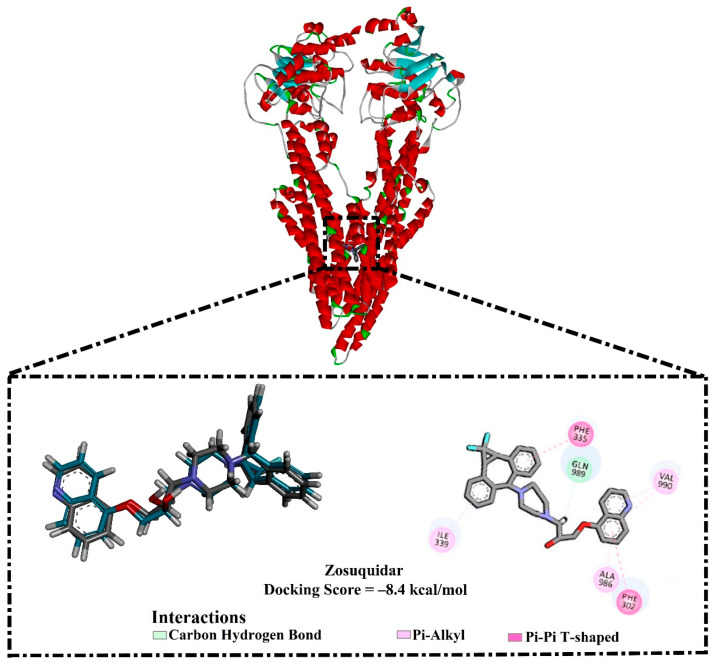
The three- and two-dimensional molecular interactions of the predicted docking pose (in cyan) and the experimental pose (in gray) for ZQU against the ABCB1 transporter.

The efficiency of AutoDock4.2.6 software in predicting the binding mode of the co-crystallized zosuquidar (ZQU) within the ABCB1 binding pocket was first assessed according to the accessible experimental data. The docking pose of ZQU was compared to its native structure (PDB ID: 6QEE [21]) (Figure 3). From Figure 3, the experimental and predicted binding modes of ZQU were matched (RMSD = 0.18 Å). ZQU exhibited a docking score with a value of −8.4 kcal/mol without forming any hydrogen bonds inside the ABCB1 binding pocket. Nevertheless, the potency of ZQU inside the ABCB1 binding pocket may be ascribed to further interactions, such as pi-pi T-shaped and carbon H-bond interactions with PHE302, PHE335, and GLN989 residues (Figure 3). By comparing the results, it was noticed that the AutoDock4.2.6 software was efficient in predicting the appropriate docking pose.

### 2.2. Virtual Screening of the T3DB Database

**Figure 4:**
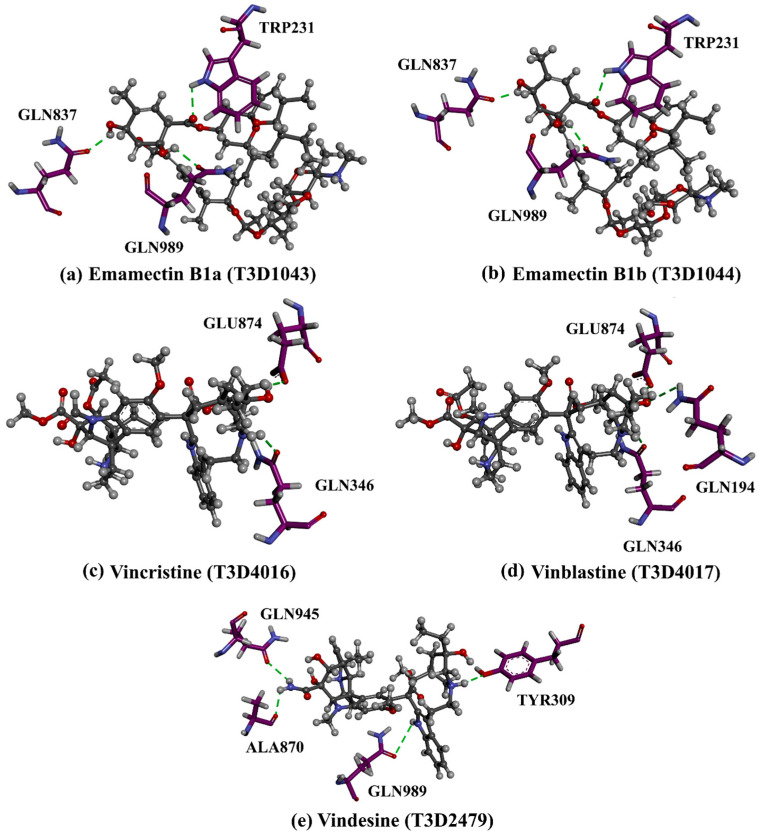
Three-dimensional molecular interactions of the predicted binding positions of (**a**) Emamectin B1a, (**b**) Emamectin B1b, (**c**) Vincristine, (**d**) Vinblastine, and (**e**) Vindesine inside the ABCB1 binding pocket. Additionally, the green dashed lines represent the conventional hydrogen bonds.

Virtual screening of large chemical databases containing hundreds of thousands of molecules could be accelerated by applying multiple filters to concentrate on molecules with drug-like characteristics [22]. However, in the current study, the T3DB database was filtered against the ABCB1 transporter based on the docking scores using AutoDock4.2.6 software. According to the estimated docking scores, only 103 T3DB compounds exhibited lower scores than that of ZQU (calc. −8.4 kcal/mol) against the ABCB1 transporter. The anticipated docking scores for the 103 T3DB compounds with the ABCB1 transporter are displayed in Table S1. Table 1 summarizes two-dimensional chemical structures, docking scores, *n*-octanol/water partition coefficient (milog*P*), and binding features of the top five potent T3DB compounds. Additionally, the three- and two-dimensional molecular interactions for these five T3DB compounds inside the ABCB1 binding pocket are depicted in Figure 4 and Figure S1, respectively. The milog*P* value, which measures permeability across the cell membrane, was less than five, indicating that these compounds have acceptable membrane permeability. Notably, these five T3DB compounds were picked out according to further binding affinity estimations throughout 10 ns MD simulations, discussed in Section 3.3.

Figure S1 implies that the investigated T3DB compounds interacted with the essential residues of the ABCB1 binding pocket, forming several hydrogen bonds, such as GLN989, GLN346, and GLU874 residues. Other interactions, such as van der Waals, hydrophobic, and pi-based interactions, were also exhibited between the identified T3DB compounds and ABCB1 transporter (Figure S1). Notably, those noncovalent interactions can have a significant contribution to the binding of the identified compound [23].

Emamectin B1a (T3D1043) and Emamectin B1b (T3D1044) belong to the family of avermectins with strong anthelmintic properties that are effective against various nematodes and arthropods [24]. Emamectin B1a and Emamectin B1b demonstrated docking scores with values of −11.8 and −12.0 kcal/mol with the ABCB1 transporter, respectively. Emamectin B1a and Emamectin B1b formed three hydrogen bonds inside the ABCB1 binding pocket (Figure 4). More specifically, the C=O group of Emamectin B1a and Emamectin B1b interacted with the NH group of TRP231 with bond lengths of 2.30 and 2.32 Å, respectively. As well, two OH groups of (3a*R*,7*S*,7a*S*)-6-methyl-2,3,7,7a-tetrahydrobenzofuran-3a,7(4*H*)-diol ring of Emamectin B1a and Emamectin B1b interacted with GLN837 and GLN989 with bond lengths of 2.04, 2.00 Å and 2.16, and 2.14 Å, respectively.

Additionally, Vincristine (T3D4016), Vinblastine (T3D4017), and Vindesine (T3D2479) belong to the family of vinca alkaloids. They are plant-derived natural products that were FDA-approved as chemotherapeutic drugs for the treatment of various cancers [25,26]. Consequently, these three drugs were repurposed towards ABCB1 transporter using in-silico computations. Vincristine, Vinblastine, and Vindesine exhibited docking scores with values of −9.9, −10.0, and −8.9 kcal/mol with the ABCB1 transporter, respectively (Table 1). For Vincristine and Vinblastine, NH and OH groups of the (1*S*,8*S*,10*S*,*Z*)-10-ethyl-1-azabicyclo[6.3.1]dodec-4-en-10-ol ring formed two hydrogen bonds with GLN346 (2.03 and 2.05 Å) and GLU874 (2.09 and 2.00 Å). For Vinblastine, the OH group of (*S*)-3-ethylpiperidin-3-ol ring formed a hydrogen bond with the NH group of GLN194 (3.20 Å).

Vindesine formed four hydrogen bonds inside the ABCB1 binding pocket with GLN945 (2.41 Å), ALA870 (2.13 Å), GLN989 (3.26 Å), and TYR309 (2.16 Å) (Figure 4). Additionally, the native structure of the ABCB1 bound to Vincristine (PDB code: 7A69 [27]) was compared to the docked structure and presented in Figure S2. From Figure S2, the docking pose was similar to the native structure of the co-crystallized Vincristine inhibitor with an RMSD value of 0.68 Å. It is worth noting that Vincristine inhibits the function of the ABCB1 transporter at high concentrations [27]. These findings proved the accuracy of the utilized in-silico approaches used in the current work.

### 2.3. Molecular Dynamics

**Figure 5:**
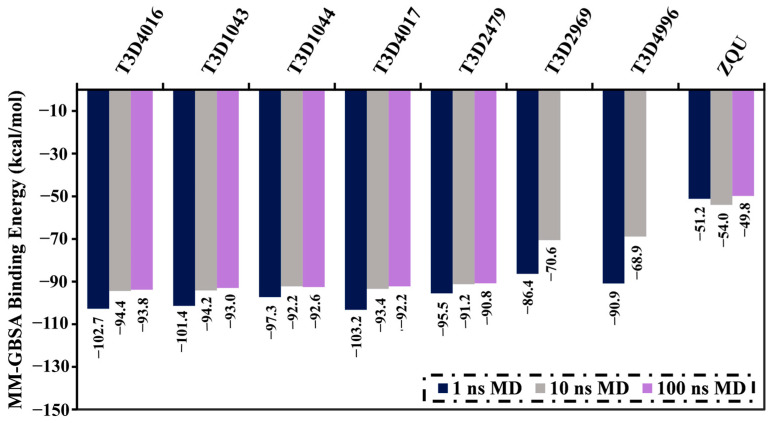
Computed binding energies of the top seven scoring toxins and ZQU inside the ABCB1 binding pocket over 1, 10, and 100 ns MD simulations.

Molecular dynamics (MD) simulations were conducted to assess and investigate the time-dependent interactions of the identified inhibitors with the ABCB1 transporter. The MD simulations were performed without considering the lipid bilayer membrane to reduce the computational cost and time. The top 103 T3DB compounds with lower binding scores than the reference ZQU (calc. −8.4 kcal/mol) complexed with the ABCB1 transporter underwent MD simulations for 1 ns. Furthermore, the corresponding MM-GBSA binding energies were evaluated (Table S1). According to the data presented in Table S1, only seven T3DB compounds demonstrated MM-GBSA binding energies less than −80.0 kcal/mol towards the ABCB1 transporter. A threshold value of −80.0 kcal/mol was chosen to shortlist the potential ABCB1 inhibitors. To gain more trustworthy results, those seven potent T3DB compounds complexed with ABCB1 transporter were subjected to longer MD simulations for 10 ns, followed by the binding affinity estimations (Figure 5). What can be inferred from Figure 5 is that five T3DB compounds, namely Emamectin B1a (T3D1043), Emamectin B1b (T3D1044), Vincristine (T3D4016), Vinblastine (T3D4017), and Vindesine (T3D2479), exhibited considerable binding energies less than −80.0 kcal/mol against the ABCB1 transporter. Further, a simulation course of 100 ns was run for those five T3DB compounds complexed with the ABCB1 transporter, and their MM-GBSA binding energies were computed (Figure 5).

It is clearly seen in Figure 5 that no substantial differences were found in the estimated binding energies of the five identified T3DB compounds over the 10 and 100 ns MD simulations. Compared to ZQU (Δ*G*_binding_ = −49.8 kcal/mol), Emamectin B1a, Emamectin B1b, Vincristine, Vinblastine, and Vindesine demonstrated promising MM-GBSA binding energies with ABCB1 transporter over the 100 ns with Δ*G*_binding_ values of −93.0, −92.6, −93.8, −92.2, and −90.8 kcal/mol, respectively. The current results shed new light on the potency of the identified compounds as promising anticancer agents.

**Figure 6:**
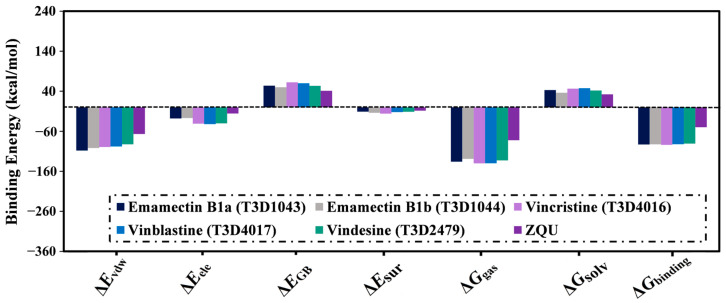
Binding affinity components of Emamectin B1a, Emamectin B1b, Vincristine, Vinblastine, Vindesine, and ZQU with the ABCB1 transporter throughout 100 ns MD simulations.

Besides, the binding energies were decomposed to grasp the forces, dominating the interaction of Emamectin B1a, Emamectin B1b, Vincristine, Vinblastine, Vindesine, and ZQU with the ABCB1 transporter (Figure 6). Based on the energy decomposition results, it was observed that the Δ*E*_vdw_ interactions significantly predominated the binding energies of Emamectin B1a, Emamectin B1b, Vincristine, Vinblastine, Vindesine, and ZQU with average values of −107.9, −101.9, −99.2, −97.8, −92.4, and −66.7 kcal/mol, respectively. The Δ*E*_ele_ interactions of Emamectin B1a, Emamectin B1b, Vincristine, Vinblastine, Vindesine, and ZQU were also favorable with average values of −28.0, −27.0, 41.0, −42.0, −40.3, and −15.6 kcal/mol, respectively.

**Figure 7:**
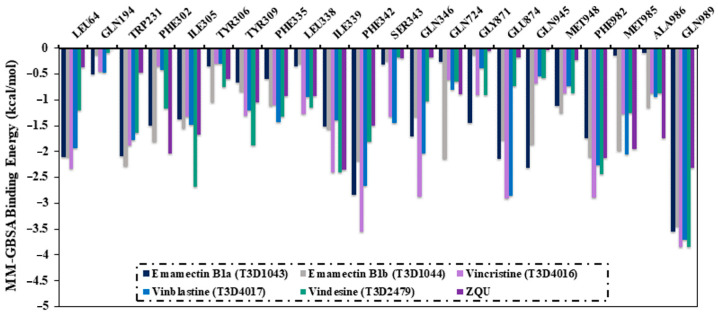
The per-residue energy decomposition of the identified T3DB compounds and ZQU with the ABCB1 transporter over 100 ns MD simulations.

To more thoroughly examine the interactions of Emamectin B1a, Emamectin B1b, Vincristine, Vinblastine, and Vindesine with the crucial residues of the ABCB1 binding pocket, the per-residue energy decomposition was executed (Figure 7). Only residues with Δ*G*_binding_ < −0.5 kcal/mol were taken into consideration. Figure 7 illustrates that PHE342, GLN346, GLU874, and GLN989 residues displayed promising contributions to the binding of the Emamectin B1a, Emamectin B1b, Vincristine, Vinblastine, and Vindesine with ABCB1 transporter. For instance, GLN989 residue exhibited −3.6, −3.4, −3.8, −3.7, −3.8, and −2.3 kcal/mol for Emamectin B1a-, Emamectin B1b-, Vincristine-, Vinblastine-, and Vindesine-, and ZQU-ABCB1 complexes, respectively.

**Figure 8:**
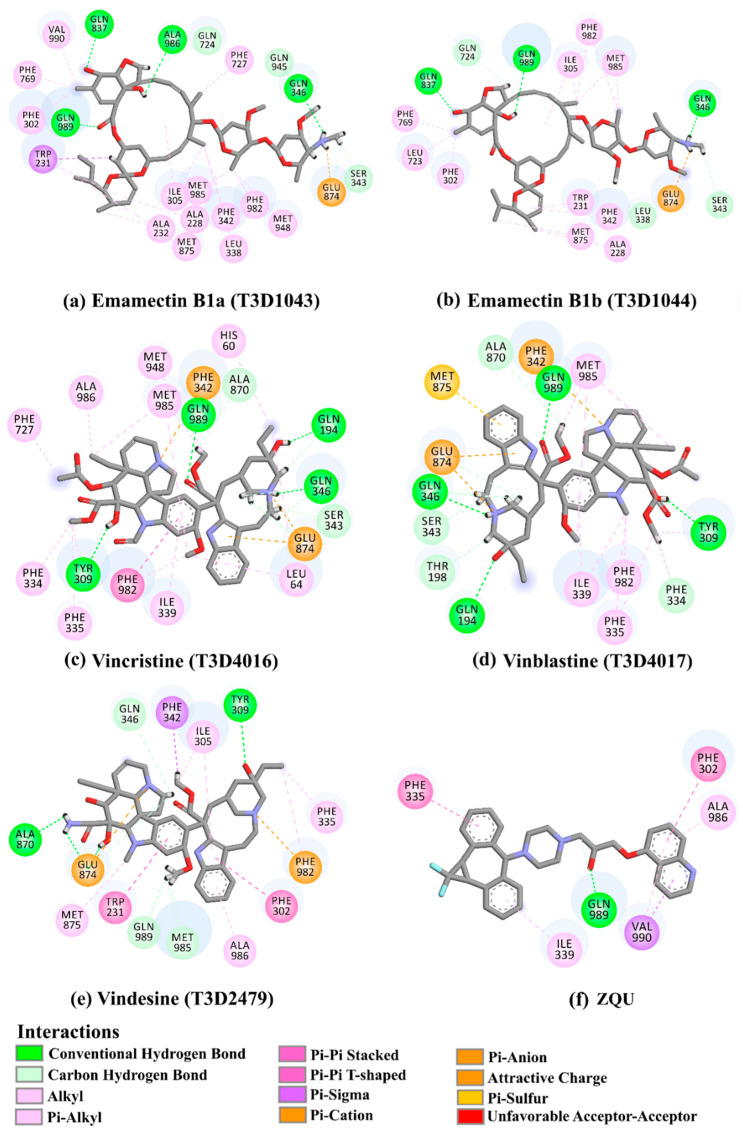
Molecular interactions of (**a**) Emamectin B1a, (**b**) Emamectin B1b, (**c**) Vincristine, (**d**) Vinblastine, (**e**) Vindesine, and (**f**) ZQU with the ABCB1 transporter according to the average structures of the 100 ns MD simulations.

Furthermore, the average structures for Emamectin B1a-, Emamectin- B1b-, Vincristine-, Vinblastine-, Vindesine-, and ZQU-ABCB1 complexes during the 100 ns MD simulations in an explicit water solvent are shown in Figure 8 and Figure S3. The top promising T3DB compounds maintained stable hydrogen bonds over the simulated time with the appearance of new bonds. For instance, Emamectin B1a showed new hydrogen bonds with ALA986 (1.75 Å) and GLN945 (1.83 Å). Remarkably, those bonds were not present in the docked structure, highlighting the necessity of executing MD simulations. Moreover, the presented results proved the significance of GLN989 residue inside the ABCB1 binding pocket as proposed by the per-residue decomposition.

### 2.4. Post-Dynamics Analyses

#### 2.4.1. Binding Energy Per-Trajectory

**Figure 9:**
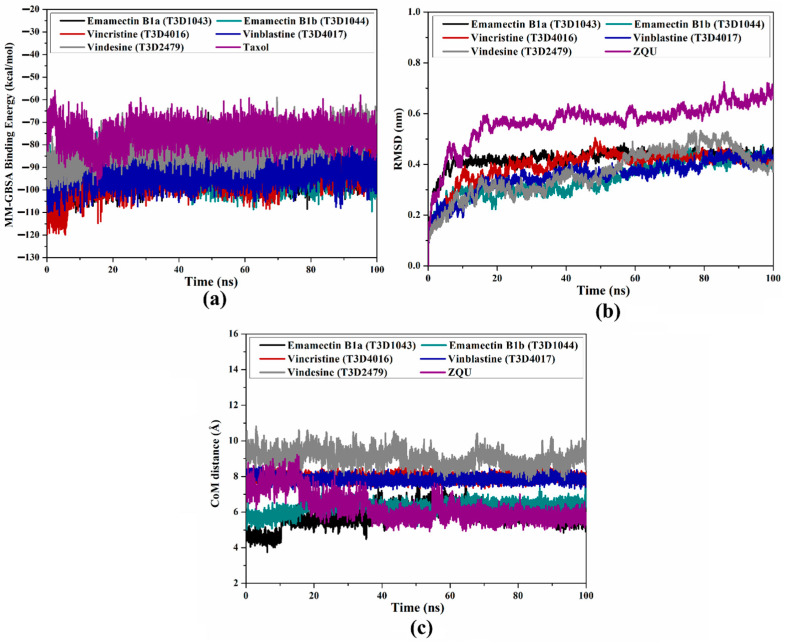
(**a**) Estimated binding energy per-trajectory, (**b**) RMSD of the backbone from the initial structure, and (**c**) CoM distances of Emamectin B1a (in black), Emamectin B1b (in cyan), Vincristine (in red), Vinblastine (in blue), Vindesine (in gray), and ZQU (in purple) complexed with the ABCB1 transporter over the 100 ns MD simulations.

The energetical constancy of Emamectin B1a, Emamectin B1b, Vincristine, Vinblastine, Vindesine, and ZQU in complex with the ABCB1 transporter was estimated by gauging the binding energy vs. time correlation (Figure 9a). From Figure 9a, it is obvious that there is outstanding stability for Emamectin B1a, Emamectin B1b, Vincristine, Vinblastine, Vindesine, and ZQU in complex with the ABCB1 transporter over the 100 ns MD simulations with average Δ*G*_binding_ values of −93.0, −92.6, −93.8, −92.2, −90.8, and −49.8 kcal/mol, respectively. These findings demonstrated the consistency of the interactions between the identified T3DB compounds complexed with the ABCB1 transporter.

#### 2.4.2. Root-Mean-Square Deviation (RMSD)

The structural variations of the identified inhibitors complexed with the ABCB1 transporter were assessed employing the RMSD analysis (Figure 9b). As shown in Figure 9b, the average RMSD values were found to be 0.42, 0.34, 0.39, 0.36, 0.37, and 0.60 Å for Emamectin B1a-, Emamectin B1b-, Vincristine-, Vinblastine-, and Vindesine-, and ZQU-ABCB1 complexes, respectively. These outcomes indicated that these inhibitors are tightly bound and generally stable inside the ABCB1 binding pocket.

#### 2.4.3. Center-of-Mass (CoM) Distance

To gain a better understanding of the stability of the identified T3DB-ABCB1 complexes, the CoM distances between the identified inhibitors and the ABCB1 transporter were examined. Figure 9c demonstrates the CoM distances between the inhibitor and GLN989 residue of the ABCB1 transporter. The average CoM distances for Emamectin B1a-, Emamectin B1b-, Vincristine-, Vinblastine-, Vindesine-, and ZQU-ABCB1 complexes were 5.8, 6.3, 8.0, 7.8, 9.0 and 6.3 Å, respectively. Notably, Emamectin B1a and B1b, Vincristine, Vinblastine, and Vindesine showed similar fluctuations to those of ZQU with the ABCB1 transporter. As a consequence, the entire stabilization of the identified inhibitors within the ABCB1 binding pocket can be deduced from these results.

#### 2.4.4. Root-Mean-Square Fluctuations (RMSF)

**Figure 10:**
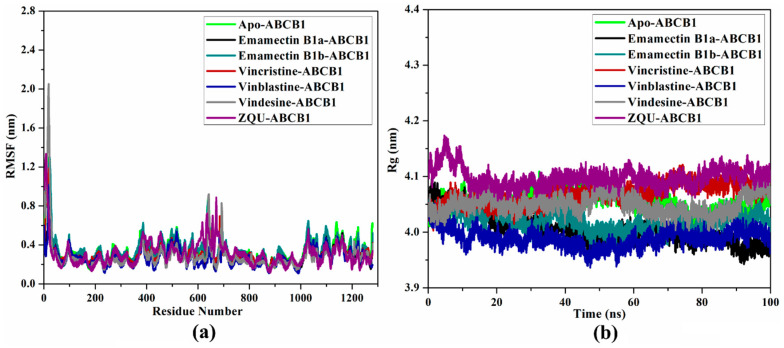
(**a**) RMSF and (**b**) Rg for apo-ABCB1 (in green), Emamectin B1a-ABCB1 (in black), Emamectin B1b-ABCB1 (in cyan), Vincristine-ABCB1 (in red), Vinblastine-ABCB1 (in blue), Vindesine-ABCB1 (in gray), and ZQU-ABCB1 (in purple) complexes over the 100 ns MD simulations.

RMSF of the alpha carbon atoms was evaluated in order to comprehend the impacts of the binding of the identified inhibitors on the structural fluctuation of the ABCB1 transporter (Figure 10a). Figure 10a elucidates that the apo-ABCB1 transporter, Emamectin B1a-, Emamectin B1b-, Vincristine-, Vinblastine-, Vindesine-, and ZQU-ABCB1 complexes exhibited average RMSF values of 0.34, 0.30, 0.35, 0.31, 0.28, 0.29, and 0.32 nm, respectively. The current outcomes indicated that the apo- and ligand-soaked structures of the ABCB1 transporter remained relatively stable.

#### 2.4.5. Radius of Gyration (Rg)

Rg was measured and plotted in Figure 10b to identify ABCB1 transporter compactness in the presence and absence of the inhibitors. The apo-, Emamectin B1a-, Emamectin B1b-, Vincristine-, Vinblastine-, Vindesine-, and ZQU-ABCB1 complexes exhibited average Rg values of 4.05, 4.01, 4.02, 4.07, 3.99, 4.05, and 4.10 nm, respectively. These results demonstrated that the ABCB1 transporter preserved its compressibility when Emamectin B1a, Emamectin B1b, Vincristine, Vinblastine, Vindesine, and ZQU were present during the simulation time.

### 2.5. Lipid Bilayer-Enhanced MD

**Figure 11:**
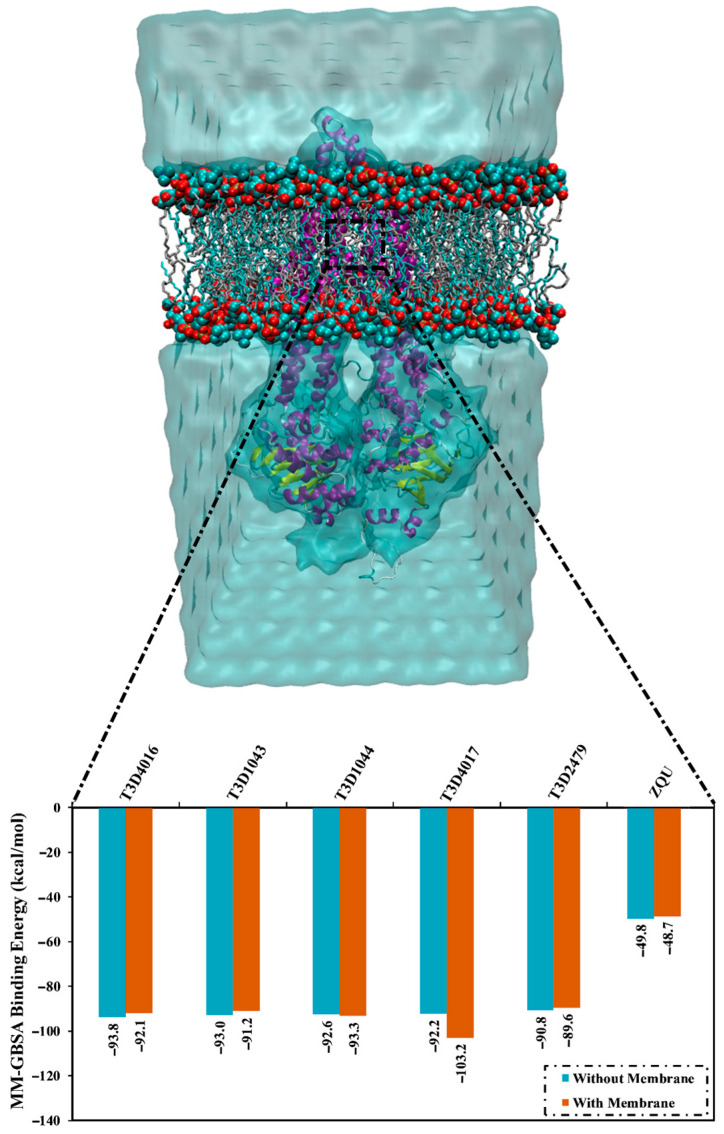
MM-GBSA binding energies for Emamectin B1a, Emamectin B1b, Vincristine, Vinblastine, Vindesine, and ZQU in the absence and presence of the POPC membrane with the ABCB1 transporter.

MD simulations can be employed to understand the transmembrane protein interface in a lipid bilayer environment [28]. Therefore, an MD simulation course of 100 ns in a POPC lipid bilayer was re-conducted for the identified inhibitors complexed with the ABCB1 transporter. The MM-GBSA binding energies were then calculated in the presence of the POPC lipid bilayer, as demonstrated in Figure 11. The presented data implies no significant differences between the two estimated values. Minutely, without the POPC membrane, the binding energies were −93.0, −92.6, −93.8, −92.2, −90.8, and −49.8 kcal/mol for Emamectin B1a, Emamectin B1b, Vincristine, Vinblastine, Vindesine, and ZQU, respectively. On the other hand, the energies of Emamectin B1a, Emamectin B1b, Vincristine, Vinblastine, Vindesine, and ZQU in the presence of the POPC were −91.2, −93.3, −92.1, −103.2, −89.6 and −48.7 kcal/mol, respectively (Figure 11).

## 3. Computational Methods

### 3.1. ABCB1 Preparation

The cryo-EM structure of the ABCB1 transporter complexed with zosuquidar (ZQU) inhibitor (PDB code: 6QEE, resolution: 3.90 Å [21]) was obtained from the PDB database and considered as a template for all computations. The ABCB1 transporter preparation included the removal of co-crystallized ligands and heteroatoms. The missing residues were constructed with the help of Modeller software [29]. States of protonation were checked using the H++ web-based server [30]. As well, the missing hydrogen atoms were inserted.

### 3.2. T3DB Database Preparation

The Toxin and Toxin-Target Database (T3DB) compounds, containing >3600 compounds, were obtained and prepared for the screening against the ABCB1 transporter [20]. All compounds were downloaded in the format of two-dimensional structural data (SDF). The duplicated compounds were excluded depending on the International Chemical Identifier (InChIKey) [31]. The three-dimensional structures were then created using Omega2 software [32,33] and subjected to energy minimization using MMFF94S implemented within SZYBKI software [34,35]. The fixpk_a_ tool implemented within the QUACPAC software was applied to scrutinize the protonation states of the inspected compounds [36]. The Gasteiger method was used to determine the partial charges of the inspected compounds [37]. The CompChem database (www.compchem.net/ccdb, accessed on 1 July 2023) provides all prepared files accessible to users.

### 3.3. Molecular Docking

In the present work, the T3DB database was filtered against the ABCB1 transporter via the AutoDock4.2.6 software [38]. The pdbqt file of the ABCB1 transporter was generated utilizing the MGL tools (version 1.5.6) [39]. The genetic algorithm (*GA*) runs, and the maximum number of energy evaluations (*eval*) variables were set to 150 and 15,000,000, respectively. Other parameters were left at their defaults. The dimensions of the grid box were set to encompass the ABCB1 binding pocket (60 Å× 60 Å × 60 Å), with a spacing value of 0.375 Å. The grid box was centered at 167.308, 170.672, and 165.91 concerning the X, Y, and Z coordinates, respectively. As well, the partition coefficient (milog*P*) of the most potent compounds was estimated using Molinspiration online server (http://www.molinspiration.com, accessed on 1 July 2023).

### 3.4. Molecular Dynamics

The docked structures of T3DB-ABCB1 complexes underwent molecular dynamics (MD) simulations using the AMBER20 software [40]. The information regarding the parameters of the MD simulations is detailed in Refs. [41,42,43,44]. Briefly, the ABCB1 transporter and the T3DB compounds were defined utilizing the AMBER force field of 14SB and the general AMBER force field (GAFF2), respectively [45,46]. The restricted electrostatic potential (RESP) approach was employed to estimate the partial charges of the T3DB compounds at the HF/6-31G* level with the help of Gaussian09 software [47,48]. Solvation of the T3DB-ABCB1 complexes took place in an octahedron box of the TIP3P model [49]. The solvated complexes were thereafter neutralized utilizing Na^+^ and Cl^–^ ions. The prepared complexes were minimized for 5000 steps before progressively heating from 0 to 310 K over 50 ps. The T3DB-ABCB1 complexes were equilibrated over 10 ns. Subsequently, the production phases were executed for 1, 10, and 100 ns within periodic boundary conditions and under an NPT ensemble. The SHAKE algorithm was used with a 2 fs integration step to constrain all bonds involving hydrogen atoms [50]. The temperature of the systems was held constant at 310 K employing a Langevin thermostat [51]. As well, the pressure was controlled through the use of Berendsen barostat [52]. The GPU version of pmemd (pmemd.cuda) within AMBER20 software was used to run all MD simulations. The CompChem GPU/CPU hybrid cluster was utilized to execute all computations (hpc.compchem.net). Finally, with the help of the Biovia Discovery Studio visualizer, all molecular interactions were displayed [53]. Besides, the conventional H-bond was defined using the default parameters implemented inside the Biovia Discovery Studio visualizer (i.e., distance 3.40 Å and angle at 120°).

### 3.5. Lipid Bilayer-Enhanced MD

The T3DB-ABCB1 complexes immersed in a lipid bilayer were constructed by the CHARMM-GUI web server [54]. For the lipid bilayer-enhanced MD simulations, a bilayer of 1-palmitoyl-2-oleoyl-phosphatidylcholine (POPC) was adopted, along with a TIP3P solvent model. The Lipid14 force field was utilized to describe the lipid bilayer [55]. Na^+^ and Cl^–^ counterions were added to neutralize the system. Besides, MD simulations of the created systems were run using the same standard parameters stated in the former section.

### 3.6. MM-GBSA Binding Energy

Utilizing the molecular mechanical-generalized Born surface area (MM-GBSA) approach, the binding energies (Δ*G*_binding_) of the investigated T3DB compounds with the ABCB1 transporter were calculated [56]. The modified GB model (igb = 2) was also used [57]. For the MM-GBSA computations, the snapshots were assembled individually every 10 ps during the production stages. The Δ*G*_binding_ was estimated as follows:

∆*G*_binding_ = *G*_Complex_ − (*G*_T3DB_ + *G*_ABCB1_) (1)

The *G* term was driven mathematically through:

*G* = *G*_GB_ + *G*_SA_ + *E*_ele_ + *E*_vdw_ (2)

where *G*_GB_, *G*_SA_, *E*_ele_, and *E*_vdw_ are solvation-free energy, nonpolar solvation-free energy, electrostatic, and van der Waals forces, respectively. Due to the cost of computations, the contributions of the configurational entropy (*S*) were ignored [58,59]. It is also important to mention that the lipid bilayer was considered in the binding energy computations.

## 4. Conclusions

The ABCB1 transporter plays an important role in the effluxion of the chemotherapeutic agents outside the targeted cell; therefore, it is considered a charming target for reversing MDR phenomena in cancer treatment. For this purpose, the T3DB database was herein mined to identify novel ABCB1 inhibitors using molecular docking, MD simulations, and MM-GBSA binding energy estimations. According to the results, Emamectin B1a (T3D1043), Emamectin B1b (T3D1044), Vincristine (T3D4016), Vinblastine (T3D4017), and Vindesine (T3D2479) exhibited promising binding affinities against the ABCB1 transporter over the 100 ns MD simulations with Δ*G*_binding_ values of −93.0, −92.6, −93.8, −92.2, and −90.8 kcal/mol, compared to ZQU (calc. −49.8 kcal/mol). The energetic and structural investigations ensured the constancy of the identified inhibitors inside the ABCB1 transporter. Additionally, the POPC simulations showed no significant difference in the binding energies of the identified inhibitors with the ABCB1 transporter. These results suggested that subsequent in-vivo and in-vitro investigations on these identified T3BD compounds may provide potential ABCB1 inhibitors that can reverse MDR phenomena.

## Figures and Tables

**Table 1:** Two-dimensional chemical structures, predicted docking scores (in kcal/mol), binding features, and origin for the top five scoring T3DB compounds and ZQU inside the ABCB1 transporter.

No.	Compound Name/Code	Origin ^a^	miLog *P*	Two-Dimensional Chemical Structures	Docking Score (kcal/mol)	Binding Features
	ZQU	------	4.9	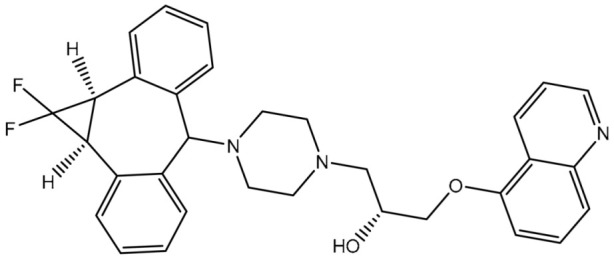	−8.4	PHE302 (Pi-Pi T-shaped, 5.07 Å), PHE335 (Pi-Pi T-shaped, 5.27 Å), GLN989 (Carbon H-bond, 2.72 Å)
1	T3D1044 (Emamectin B1b)	Bacterial toxin (Streptomyces avermitilis)	2.3	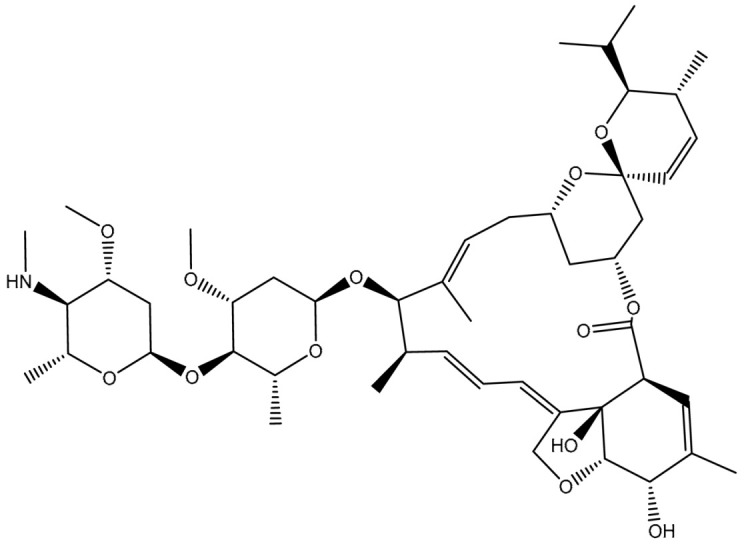	−12.0	TRP231 (Conventional H-bond, 2.32 Å; Pi-Sigma, 2.72 Å), TYR309 (Carbon H-bond, 2.97 Å), ILE339 (Carbon H-bond, 2.97, 3.26 Å), SER343 (Carbon H-bond, 2.61 Å), GLN837 (Conventional H-bond, 2.00 Å), GLN989 (Conventional H-bond, 2.14 Å; Carbon H-bond, 2.62 Å)
2	T3D1043 (Emamectin B1a)	Bacterial toxin (Streptomyces avermitilis)	2.8	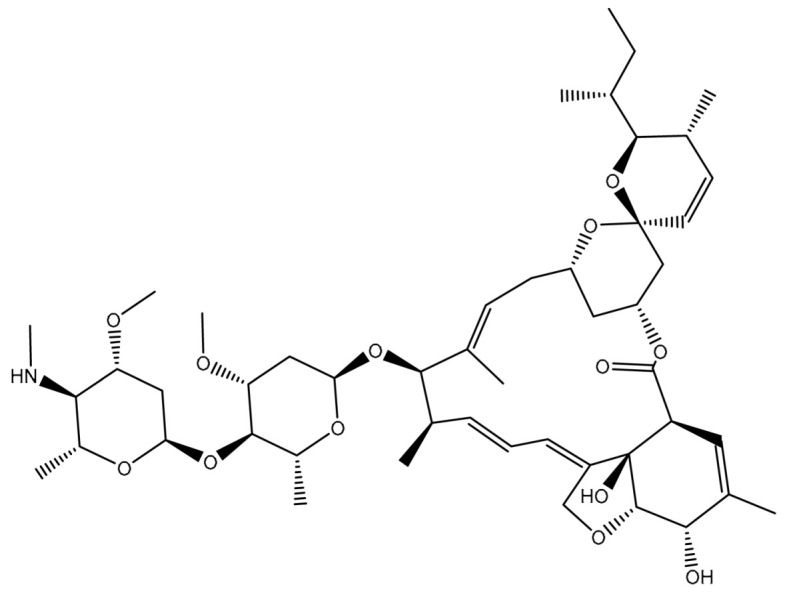	−11.8	TRP231 (Conventional H-bond, 2.30 Å; Pi-Sigma, 2.70 Å), GLN837 (Conventional H-bond, 2.04 Å), GLN989 (Conventional H-bond, 2.16 Å; Carbon H-bond, 2.67 Å), SER343 (Carbon H-bond, 2.34 Å), ILE339 (Carbon H-bond, 2.70, 2.80 Å)
3	T3D4017 (Vinblastine)	Synthetic compound (treatment of breast cancer)	5.6	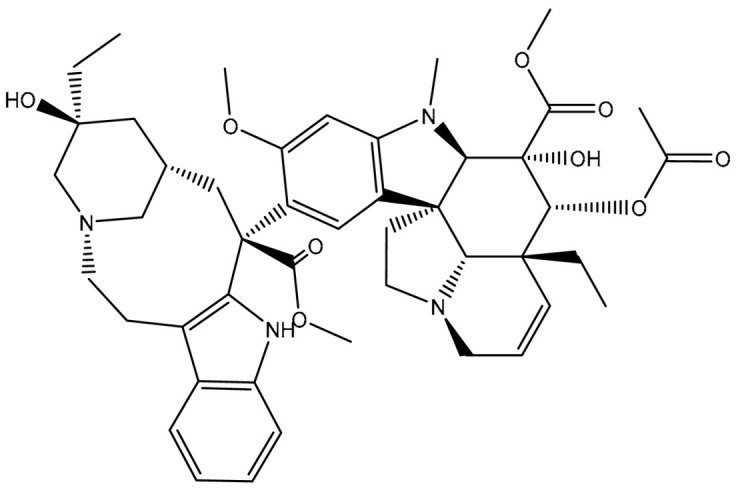	−10.0	GLN194 (Conventional H-bond, 3.20 Å), TRP231 (Pi-Pi Stacked, 4.64 Å), PHE342 (Pi-Pi Stacked, 4.58, 5.88 Å), GLN346 (Conventional H-bond, 2.05 Å; Carbon H-bond, 2.91 Å), GLU874 (Conventional H-bond, 2.00 Å; Carbon H-bond, 2.46, 3.06, 3.11 Å; Attractive charge, 4.26 Å)
4	T3D4016 (Vincristine)	Synthetic compound (treatment of acute lymphocytic leukemia)	4.9	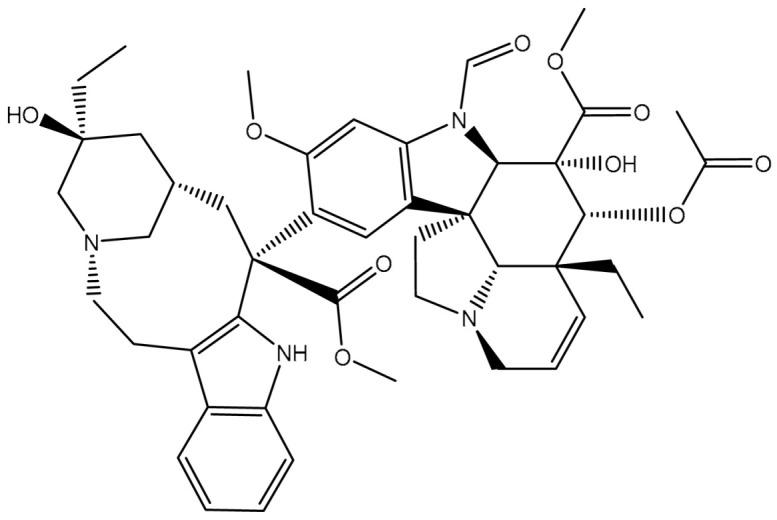	−9.9	TRP231 (Pi-Pi Stacked, 4.62 Å), PHE342 (Pi-Pi Stacked, 4.49, 5.71 Å), SER343 (Carbon H-bond, 2.49 Å), GLN346 (Conventional H-bond, 2.03 Å; Carbon H-bond, 2.93 Å), GLU874 (Conventional H-bond, 2.09 Å; Carbon H-bond, 2.61, 2.86 Å; Attractive charge, 4.38 Å)
5	T3D2479 (Vindesine)	Synthetic compound (antineoplastic agent)	3.7	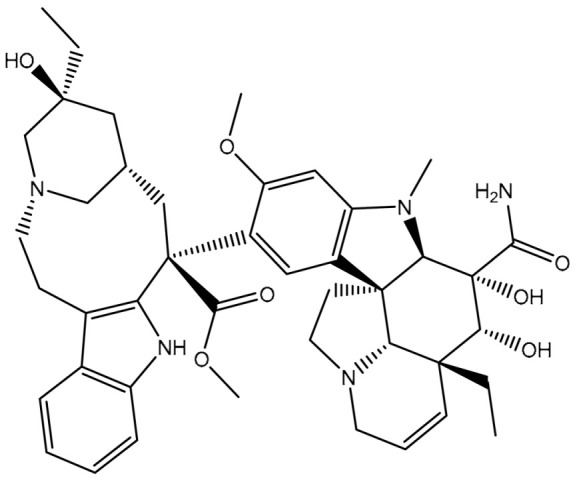	−8.9	TYR309 (Conventional H-bond, 2.16 Å), ALA870 (Conventional H-bond, 2.13 Å), GLY871 (Carbon H-bond, 2.99 Å), GLU874 (Carbon H-bond, 1.85, 2.74 Å; Attractive charge, 3.57 Å), GLN945 (Conventional H-bond, 2.41 Å), MET985 (Carbon H-bond, 2.83 Å), GLN989 (Conventional H-bond, 3.26 Å)

^a^ Taken from the T3DB online database [20].

## Data Availability

The data presented in this study are available in the Supplementary Materials.

## Supplementary Materials

The following supporting information can be downloaded at: https://www.mdpi.com/article/10.3390/ph16071019/s1, Figure S1: 2D molecular interactions of the top five T3DB compounds inside the ABCB1 binding pocket; Figure S2: (a) 3D representation of the predicted docking pose (in cyan) and the experimental pose (in gray), and 2D representation of interactions of the experimental pose of Vincristine inside the ABCB1 binding pocket. The docking score is also presented (in kcal/mol). Figure S3: 3D molecular interactions of the top five T3DB compounds and ZQU inside the ABCB1 binding pocket according to the average structures over 100 ns MD simulations (the hydrogen bonds are represented in green dashed lines); Table S1: Estimated docking scores and MM-GBSA binding energies (in kcal/mol) over 1 ns for the top 103 T3DB compounds compared to ZQU inside the ABCB1 binding pocket.

## References

[1] Debela D.T., Muzazu S.G., Heraro K.D., Ndalama M.T., Mesele B.W., Haile D.C., Kitui S.K., Manyazewal T. (2021). New approaches and procedures for cancer treatment: Current perspectives. SAGE Open Med..

[2] Gottesman M.M., Ling V. (2006). The molecular basis of multidrug resistance in cancer: The early years of P-glycoprotein research. FEBS J..

[3] Wu Q., Yang Z., Nie Y., Shi Y., Fan D. (2014). Multi-drug resistance in cancer chemotherapeutics: Mechanisms and lab approaches. Cancer Lett..

[4] Eckford P.D., Sharom F.J. (2009). ABC efflux pump-based resistance to chemotherapy drugs. Chem. Rev..

[5] Szakacs G., Paterson J.K., Ludwig J.A., Booth-Genthe C., Gottesman M.M. (2006). Targeting multidrug resistance in cancer. Nat. Rev. Drug Discov..

[6] Dean M., Annilo T. (2005). Evolution of the ATP-binding cassette (ABC) transporter superfamily in vertebrates. Annu. Rev. Genom. Hum. Genet..

[7] Ween M.P., Armstrong M.A., Oehler M.K., Ricciardelli C. (2015). The role of ABC transporters in ovarian cancer progression and chemoresistance. Crit. Rev. Oncol. Hematol..

[8] Sharom F.J. (2008). ABC multidrug transporters: Structure, function and role in chemoresistance. Pharmacogenomics.

[9] Lage H. (2008). An overview of cancer multidrug resistance: A still unsolved problem. Cell. Mol. Life Sci..

[10] Chen K.G., Sikic B.I. (2012). Molecular pathways: Regulation and therapeutic implications of multidrug resistance. Clin. Cancer Res..

[11] Juliano R.L., Ling V. (1976). A surface glycoprotein modulating drug permeability in Chinese hamster ovary cell mutants. Biochim. Biophys. Acta.

[12] Thiebaut F., Tsuruo T., Hamada H., Gottesman M.M., Pastan I., Willingham M.C. (1987). Cellular localization of the multidrug-resistance gene product P-glycoprotein in normal human tissues. Proc. Natl. Acad. Sci. USA.

[13] Gutmann D.A., Ward A., Urbatsch I.L., Chang G., van Veen H.W. (2010). Understanding polyspecificity of multidrug ABC transporters: Closing in on the gaps in ABCB1. Trends Biochem. Sci..

[14] Wu C.P., Calcagno A.M., Ambudkar S.V. (2008). Reversal of ABC drug transporter-mediated multidrug resistance in cancer cells: Evaluation of current strategies. Curr. Mol. Pharmacol..

[15] Crowley E., McDevitt C.A., Callaghan R., Zhou J. (2010). Generating Inhibitors of P-Glycoprotein: Where to, Now?. Multi-Drug Resistance in Cancer.

[16] Harvey A.L. (2014). Toxins and drug discovery. Toxicon.

[17] Bordon K.C.F., Cologna C.T., Fornari-Baldo E.C., Pinheiro-Junior E.L., Cerni F.A., Amorim F.G., Anjolette F.A.P., Cordeiro F.A., Wiezel G.A., Cardoso I.A. (2020). From Animal Poisons and Venoms to Medicines: Achievements, Challenges and Perspectives in Drug Discovery. Front. Pharmacol..

[18] Bharadwaj S., Dubey A., Kamboj N.K., Sahoo A.K., Kang S.G., Yadava U. (2021). Drug repurposing for ligand-induced rearrangement of Sirt2 active site-based inhibitors via molecular modeling and quantum mechanics calculations. Sci. Rep..

[19] Radwan M.O., Ciftci H.I., Ali T.F.S., Ellakwa D.E., Koga R., Tateishi H., Nakata A., Ito A., Yoshida M., Okamoto Y. (2019). Antiproliferative S-Trityl-l-Cysteine-Derived Compounds as SIRT2 Inhibitors: Repurposing and Solubility Enhancement. Molecules.

[20] Wishart D., Arndt D., Pon A., Sajed T., Guo A.C., Djoumbou Y., Knox C., Wilson M., Liang Y., Grant J. (2015). T3DB: The toxic exposome database. Nucleic Acids Res..

[21] Alam A., Kowal J., Broude E., Roninson I., Locher K.P. (2019). Structural insight into substrate and inhibitor discrimination by human P-glycoprotein. Science.

[22] Kralj S., Jukič M., Bren U. (2022). Comparative Analyses of Medicinal Chemistry and Cheminformatics Filters with Accessible Implementation in Konstanz Information Miner (KNIME). Int. J. Mol. Sci..

[23] Lesnik S., Jukic M., Bren U. (2023). Mechanistic Insights of Polyphenolic Compounds from Rosemary Bound to Their Protein Targets Obtained by Molecular Dynamics Simulations and Free-Energy Calculations. Foods.

[24] Burg R.W., Miller B.M., Baker E.E., Birnbaum J., Currie S.A., Hartman R., Kong Y.L., Monaghan R.L., Olson G., Putter I. (1979). Avermectins, new family of potent anthelmintic agents: Producing organism and fermentation. Antimicrob. Agents Chemother..

[25] Martino E., Casamassima G., Castiglione S., Cellupica E., Pantalone S., Papagni F., Rui M., Siciliano A.M., Collina S. (2018). Vinca alkaloids and analogues as anti-cancer agents: Looking back, peering ahead. Bioorg. Med. Chem. Lett..

[26] Zhang Y.-W., Kong X.-Y., Wang J.-H., Du G.-H., Du G.-H. (2018). Vinblastine and Vincristine. Natural Small Molecule Drugs from Plants.

[27] Nosol K., Romane K., Irobalieva R.N., Alam A., Kowal J., Fujita N., Locher K.P. (2020). Cryo-EM structures reveal distinct mechanisms of inhibition of the human multidrug transporter ABCB1. Proc. Natl. Acad. Sci. USA.

[28] Stansfeld P.J., Sansom M.S. (2011). Molecular simulation approaches to membrane proteins. Structure.

[29] Marti-Renom M.A., Stuart A.C., Fiser A., Sanchez R., Melo F., Sali A. (2000). Comparative protein structure modeling of genes and genomes. Annu. Rev. Biophys. Biomol. Struct..

[30] Gordon J.C., Myers J.B., Folta T., Shoja V., Heath L.S., Onufriev A. (2005). H++: A server for estimating pKas and adding missing hydrogens to macromolecules. Nucleic Acids Res..

[31] Heller S.R., McNaught A., Pletnev I., Stein S., Tchekhovskoi D. (2015). InChI, the IUPAC International Chemical Identifier. J. Cheminformatics.

[32] (2013). OMEGA.

[33] Hawkins P.C., Skillman A.G., Warren G.L., Ellingson B.A., Stahl M.T. (2010). Conformer generation with OMEGA: Algorithm and validation using high quality structures from the Protein Databank and Cambridge Structural Database. J. Chem. Inf. Model..

[34] (2016). SZYBKI.

[35] Halgren T.A. (1999). MMFF VI. MMFF94s option for energy minimization studies. J. Comput. Chem..

[36] (2016). QUACPAC.

[37] Gasteiger J., Marsili M. (1980). Iterative partial equalization of orbital electronegativity—A rapid access to atomic charges. Tetrahedron.

[38] Morris G.M., Huey R., Lindstrom W., Sanner M.F., Belew R.K., Goodsell D.S., Olson A.J. (2009). AutoDock4 and AutoDockTools4: Automated docking with selective receptor flexibility. J. Comput. Chem..

[39] Forli S., Huey R., Pique M.E., Sanner M.F., Goodsell D.S., Olson A.J. (2016). Computational protein-ligand docking and virtual drug screening with the AutoDock suite. Nat. Protoc..

[40] Case D.A., Betz R.M., Cerutti D.S., Cheatham T.E., Darden T.A., Duke R.E., Giese T.J., Gohlke H., Goetz A.W., Homeyer N. (2016). AMBER.

[41] Ibrahim M.A.A., Abdeljawaad K.A.A., Abdelrahman A.H.M., Jaragh-Alhadad L.A., Oraby H.F., Elkaeed E.B., Mekhemer G.A.H., Gabr G.A., Shawky A.M., Sidhom P.A. (2022). Exploring natural product activity and species source candidates for hunting ABCB1 transporter inhibitors: An in silico drug discovery study. Molecules.

[42] Ibrahim M.A.A., Abdelrahman A.H.M., Badr E.A.A., Almansour N.M., Alzahrani O.R., Ahmed M.N., Soliman M.E.S., Naeem M.A., Shawky A.M., Sidhom P.A. (2022). Naturally occurring plant-based anticancerous candidates as prospective ABCG2 inhibitors: An in silico drug discovery study. Mol. Divers..

[43] Ibrahim M.A.A., Badr E.A.A., Abdelrahman A.H.M., Almansour N.M., Mekhemer G.A.H., Shawky A.M., Moustafa M.F., Atia M.A.M. (2022). In Silico targeting human multidrug transporter ABCG2 in breast cancer: Database screening, molecular docking, and molecular dynamics study. Mol. Inform..

[44] Ibrahim M.A.A., Abdelrahman A.H.M., Jaragh-Alhadad L.A., Atia M.A.M., Alzahrani O.R., Ahmed M.N., Moustafa M.S., Soliman M.E.S., Shawky A.M., Pare P.W. (2022). Exploring Toxins for Hunting SARS-CoV-2 Main Protease Inhibitors: Molecular Docking, Molecular Dynamics, Pharmacokinetic Properties, and Reactome Study. Pharmaceuticals.

[45] Maier J.A., Martinez C., Kasavajhala K., Wickstrom L., Hauser K.E., Simmerling C. (2015). ff14SB: Improving the accuracy of protein side chain and backbone parameters from ff99SB. J. Chem. Theory Comput..

[46] Wang J., Wolf R.M., Caldwell J.W., Kollman P.A., Case D.A. (2004). Development and testing of a general amber force field. J. Comput. Chem..

[47] Bayly C.I., Cieplak P., Cornell W.D., Kollman P.A. (1993). A well-behaved electrostatic potential based method using charge restraints for deriving atomic charges—The RESP model. J. Phys. Chem..

[48] Frisch M.J.T.G.W., Schlegel H.B., Scuseria G.E., Robb M.A., Cheeseman J.R., Scalmani G., Barone V., Mennucci B., Petersson G.A., Nakatsuji H. (2009). Gaussian 09, Revision E01.

[49] Jorgensen W.L., Chandrasekhar J., Madura J.D., Impey R.W., Klein M.L. (1983). Comparison of simple potential functions for simulating liquid water. J. Chem. Phys..

[50] Miyamoto S., Kollman P.A. (1992). Settle—An Analytical Version of the Shake and Rattle Algorithm for Rigid Water Models. J. Comput. Chem..

[51] Izaguirre J.A., Catarello D.P., Wozniak J.M., Skeel R.D. (2001). Langevin stabilization of molecular dynamics. J. Chem. Phys..

[52] Berendsen H.J.C., Postma J.P.M., Vangunsteren W.F., Dinola A., Haak J.R. (1984). Molecular-dynamics with coupling to an external bath. J. Chem. Phys..

[53] Dassault Systèmes (2019). Dassault Systèmes BIOVIA, Discovery Studio Visualize, version 2019.

[54] Jo S., Kim T., Iyer V.G., Im W. (2008). CHARMM-GUI: A web-based graphical user interface for CHARMM. J. Comput. Chem..

[55] Dickson C.J., Madej B.D., Skjevik A.A., Betz R.M., Teigen K., Gould I.R., Walker R.C. (2014). Lipid14: The amber lipid force field. J. Chem. Theory Comput..

[56] Massova I., Kollman P.A. (2000). Combined molecular mechanical and continuum solvent approach (MM-PBSA/GBSA) to predict ligand binding. Perspect. Drug Discov..

[57] Onufriev A., Bashford D., Case D.A. (2004). Exploring protein native states and large-scale conformational changes with a modified generalized born model. Proteins.

[58] Hou T., Wang J., Li Y., Wang W. (2011). Assessing the performance of the molecular mechanics/Poisson Boltzmann surface area and molecular mechanics/generalized Born surface area methods. II. The accuracy of ranking poses generated from docking. J. Comput. Chem..

[59] Wang E., Sun H., Wang J., Wang Z., Liu H., Zhang J.Z.H., Hou T. (2019). End-point binding free energy calculation with MM/PBSA and MM/GBSA: Strategies and applications in drug design. Chem. Rev..

