# Supplementary material for: In-Silico Mining of the Toxins Database (T3DB) towards Hunting Prospective Candidates as ABCB1 Inhibitors: Integrated Molecular Docking and Lipid Bilayer-Enhanced Molecular Dynamics Study
Source: Pharmaceuticals (Basel). 2023 Jul 18;16(7):1019. doi: 10.3390/ph16071019 (PMC10384459; doi:10.3390/ph16071019)
Supplement: Supplementary file 1 [file pharmaceuticals-16-01019-s001.zip › pharmaceuticals-2473993-supplementary.pdf]

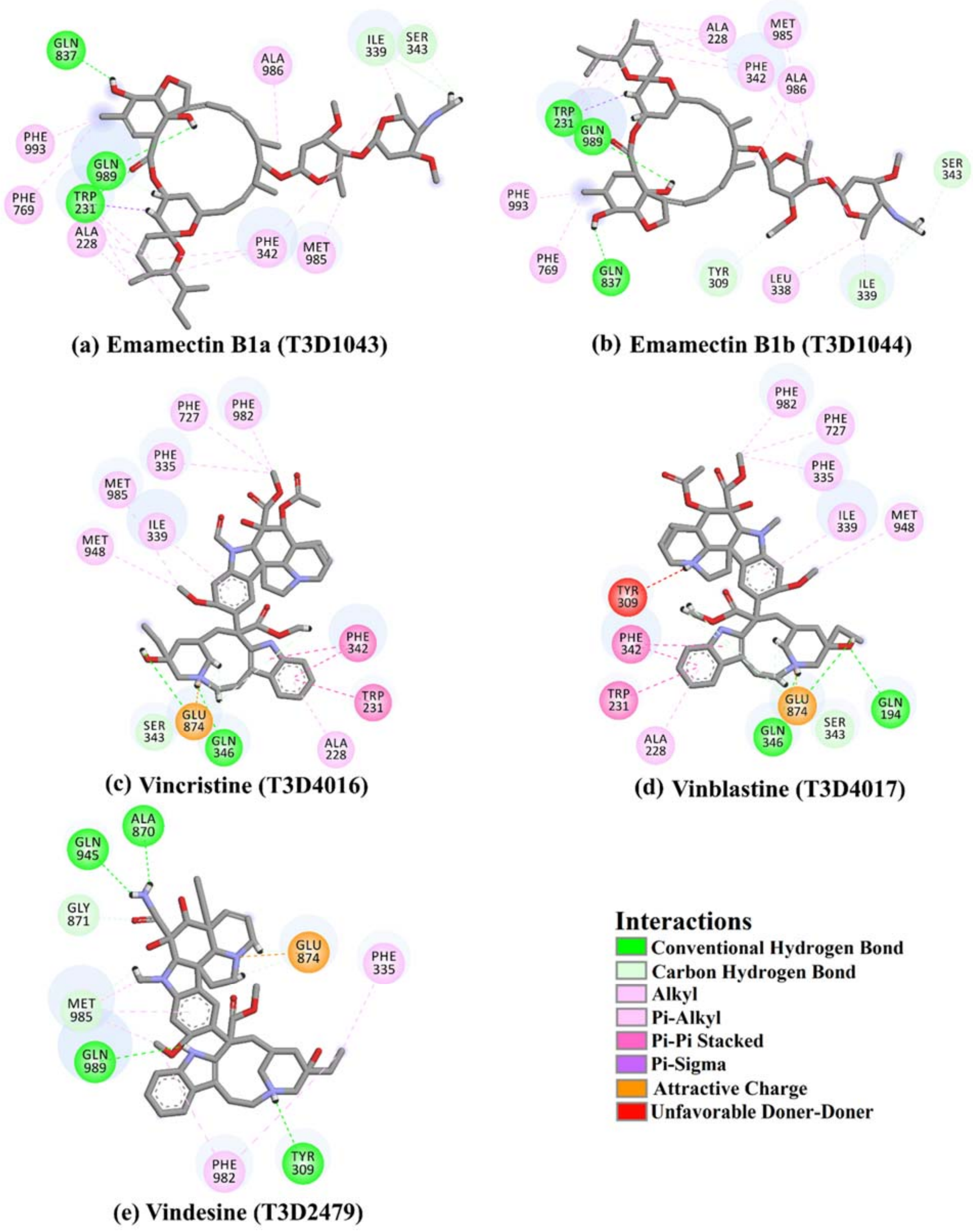

Figure S1. 2D molecular interactions of the top five T3DB compounds inside the ABCB1 binding pocket.

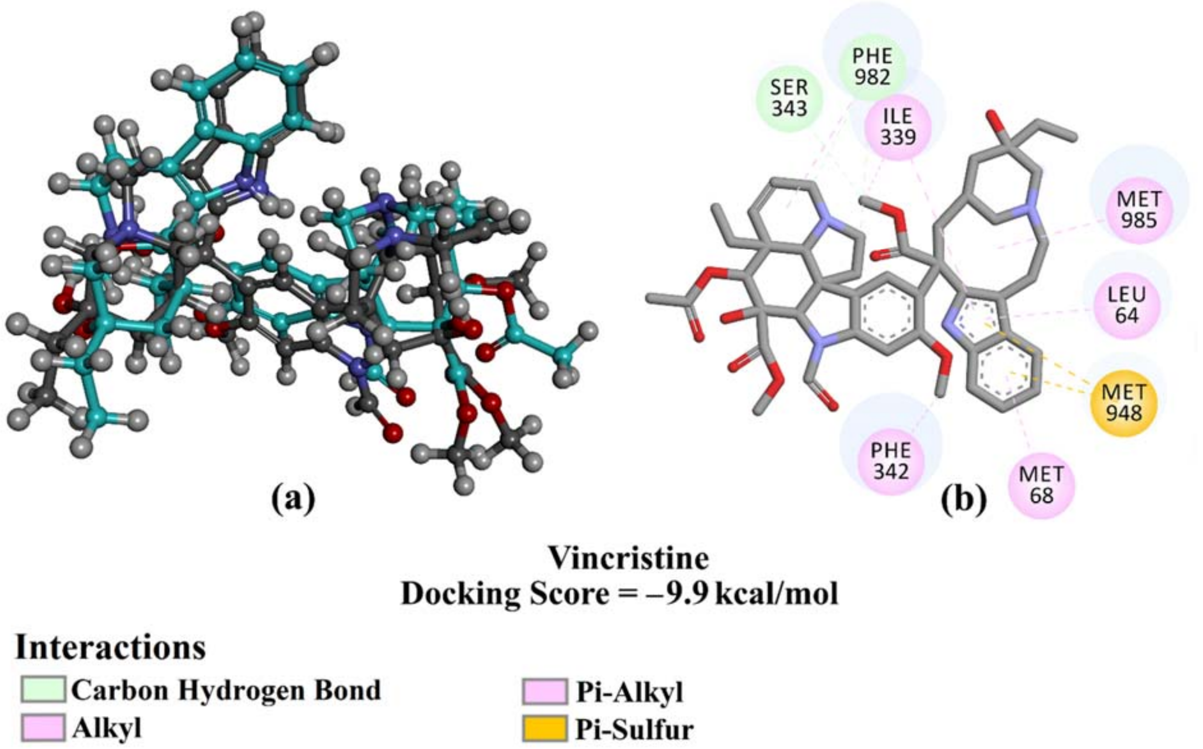

Figure S2. (a) 3D representation of the predicted docking pose (in cyan) and the experimental pose (in gray), and 2D representation of interactions of the experimental pose of Vincristine inside the ABCB1 binding pocket. The docking score is also presented (in kcal/mol).

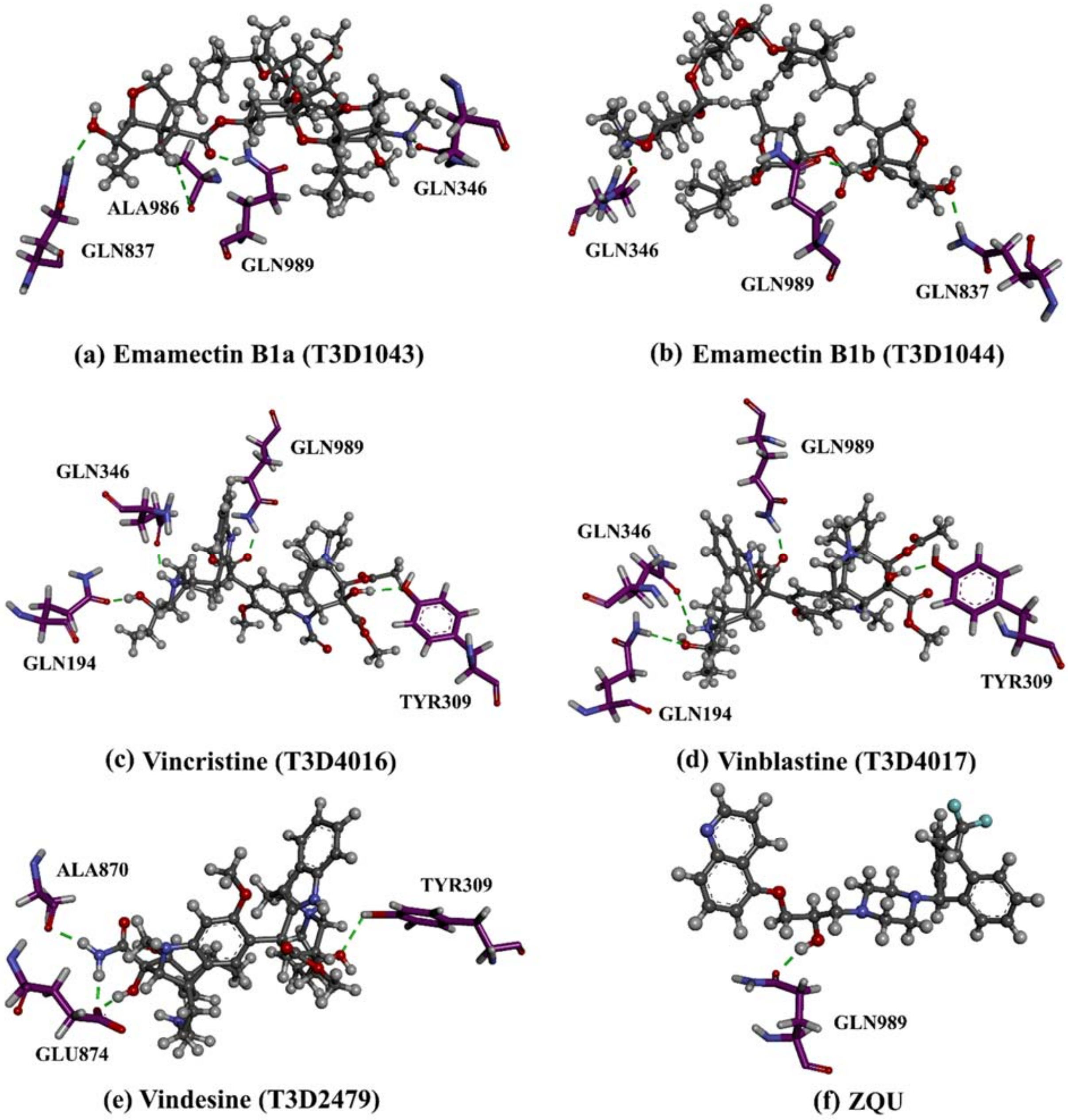

Figure S3. 3D molecular interactions of the top five T3DB compounds and ZQU inside the ABCB1 binding pocket according to the average structures over 100 ns MD simulations (the hydrogen bonds are represented in green dashed lines).

Table S1. Estimated docking scores and MM-GBSA binding energies (in kcal/mol) over 1 ns for the top 103 T3DB compounds compared to ZQU inside the ABCB1 binding pocket. [a]

No.	Toxin Code	Docking Score (kcal/mol)	MM-GBSA Binding Energy (kcal/mol)
	**ZQU**	**–8.4**	**–51.2**
1	T3D4017	–10.0	–108.2
2	T3D4016	–9.9	–102.7
3	T3D1043	–11.8	–101.4
4	T3D1044	–12.0	–97.3
5	T3D2479	–8.9	–95.5
6	T3D4996	–12.3	–90.9
7	T3D2969	–9.9	–86.4
8	T3D2838	–9.2	–78.2
9	T3D2672	–12.7	–77.1
10	T3D4021	–9.6	–77.5
11	T3D4995	–13.2	–76.0
12	T3D4323	–10.7	–73.5
13	T3D2670	–9.0	–72.3
14	T3D2727	–9.7	–72.2
15	T3D4019	–9.9	–70.7
16	T3D2750	–9.7	–70.0
17	T3D3740	–11.7	–68.5
18	T3D4916	–11.1	–68.5
19	T3D4084	–10.1	–68.3
20	T3D4440	–9.7	–68.1
21	T3D4438	–9.4	–66.7
22	T3D2460	–9.5	–62.4
23	T3D3887	–9.9	–61.7
24	T3D2959	–9.6	–60.7
25	T3D4439	–9.9	–59.6
26	T3D2871	–8.6	–58.5
27	T3D2978	–9.4	–58.1
28	T3D2713	–9.2	–56.9
29	T3D2576	–9.5	–56.7
30	T3D4235	–9.4	–56.0
31	T3D2984	–9.4	–55.8
32	T3D4067	–8.5	–55.2
33	T3D4083	–9.9	–54.8
34	T3D2907	–8.9	–54.1
35	T3D2703	–8.9	–52.7
36	T3D2677	–8.8	–52.5
37	T3D2043	–8.8	–52.4
38	T3D4082	–9.1	–52.1
39	T3D2974	–10.3	–51.8
40	T3D2044	–8.9	–47.4
41	T3D2358	–9.0	–47.3
42	T3D2143	–9.2	–46.9
43	T3D2692	–9.5	–45.6
44	T3D2697	–9.4	–45.4
45	T3D2015	–9.1	–45.2
46	T3D2852	–8.6	–44.8
47	T3D2011	–9.0	–44.6
48	T3D2008	–9.3	–44.2

Table S1. *Continued.*

No.	Toxin Code	Docking Score (kcal/mol)	MM-GBSA Binding Energy (kcal/mol)
49	T3D2012	–9.4	–43.6
50	T3D2134	–8.5	–43.5
51	T3D2483	–9.9	–43.3
52	T3D2006	–8.6	–42.6
53	T3D2680	–8.7	–42.6
54	T3D2041	–8.6	–42.3
55	T3D0631	–9.3	–42.1
56	T3D2234	–8.7	–41.9
57	T3D4910	–8.5	–41.3
58	T3D2007	–8.7	–41.2
59	T3D3895	–8.5	–40.9
60	T3D2164	–8.5	–40.9
61	T3D0588	–8.5	–40.3
62	T3D2017	–8.9	–40.2
63	T3D2139	–8.6	–40.1
64	T3D2917	–8.7	–39.6
65	T3D2228	–8.5	–39.2
66	T3D2324	–8.6	–39.1
67	T3D2001	–8.7	–39.1
68	T3D2162	–8.6	–39.0
69	T3D2796	–8.5	–38.8
70	T3D0571	–8.6	–37.9
71	T3D2753	–9.0	–37.1
72	T3D4240	–8.7	–36.9
73	T3D4283	–8.7	–36.8
74	T3D0556	–8.8	–36.7
75	T3D2014	–8.9	–36.6
76	T3D3875	–8.8	–35.8
77	T3D0546	–8.5	–35.8
78	T3D4282	–9.6	–35.6
79	T3D2010	–8.6	–35.5
80	T3D0557	–8.8	–35.3
81	T3D0580	–8.9	–34.7
82	T3D0632	–9.2	–34.3
83	T3D2009	–8.6	–34.1
84	T3D2158	–8.5	–33.9
85	T3D0542	–8.5	–33.8
86	T3D0512	–8.6	–33.7
87	T3D0569	–8.8	–33.6
88	T3D2003	–8.6	–33.3
89	T3D0526	–8.6	–32.6
90	T3D4923	–9.7	–31.5
91	T3D4242	–8.6	–31.2
92	T3D0015	–8.8	–30.9
93	T3D0547	–8.5	–30.0
94	T3D0174	–8.7	–29.3
95	T3D0009	–8.8	–28.7
96	T3D0636	–9.2	–28.4
97	T3D2825	–9.3	–27.9
98	T3D0629	–9.0	–27.7
99	T3D0622	–8.7	–27.6
100	T3D2012	–9.4	–43.6

Table S1. *Continued.*

No.	Toxin Code	Docking Score (kcal/mol)	MM-GBSA Binding Energy (kcal/mol)
101	T3D0070	–8.5	–25.9
102	T3D0010	–8.5	–25.2
103	T3D0062	–8.5	–23.6

[a] Data ranked based on the MM-GBSA binding energy over the 1 ns MD simulations.
